# Was an increase in cocaine use among injecting drug users in New South Wales, Australia, accompanied by an increase in violent crime?

**DOI:** 10.1186/1471-2458-5-40

**Published:** 2005-04-19

**Authors:** Louisa Degenhardt, Carolyn Day, Wayne Hall, Elizabeth Conroy, Stuart Gilmour

**Affiliations:** 1 National Drug and Alcohol Research Centre, University of New South Wales, Sydney Australia; 2 National Centre in HIV Epidemiology & Clinical Research, University of New South Wales, Sydney Australia; 3 Office of Public Policy and Ethics, Institute for Molecular Bioscience, University of Queensland, St Lucia Australia

## Abstract

**Background:**

A sharp reduction in heroin supply in Australia in 2001 was followed by a large but transient increase in cocaine use among injecting drug users (IDU) in Sydney. This paper assesses whether the increase in cocaine use among IDU was accompanied by increased rates of violent crime as occurred in the United States in the 1980s. Specifically, the paper aims to examine the impact of increased cocaine use among Sydney IDU upon police incidents of robbery with a weapon, assault and homicide.

**Methods:**

Data on cocaine use among IDU was obtained from the Illicit Drug Reporting System (IDRS). Monthly NSW Police incident data on arrests for cocaine possession/use, robbery offences, homicides, and assaults, were obtained from the Bureau of Crime Statistics and Research. Time series analysis was conducted on the police data series where possible. Semi-structured interviews were conducted with representatives from law enforcement and health agencies about the impacts of cocaine use on crime and policing.

**Results:**

There was a significant increase in cocaine use and cocaine possession offences in the months immediately following the reduction in heroin supply. There was also a significant increase in incidents of robbery where weapons were involved. There were *no *increases in offences involving firearms, homicides or reported assaults.

**Conclusion:**

The increased use of cocaine among injecting drug users following the heroin shortage led to increases in violent crime. Other States and territories that also experienced a heroin shortage but did not show any increases in cocaine use did not report any increase in violent crimes. The violent crimes committed did not involve guns, most likely because of its stringent gun laws, in contrast to the experience of American cities that have experienced high rates of cocaine use and violent crime.

## Background

In the United States in the mid and late 1980s, there was a cocaine epidemic fuelled by the use of "crack" cocaine [1-4]. At the same time, increases were noted in violent crime [5]. New York experienced a particularly notable increase in the extent of violent crime in the city [4,6,7]. These violent crimes often involved firearms and led to an increased homicide rate [5]. More recently, increases in the availability and use of crack in the United Kingdom (UK) [8,9] have also been accompanied by increases in violent crime, also involving firearms [10].

Goldstein [11] proposed a tripartite model to explain why crime may be related to drug use. First, the psychopharmacology of the drug may increase the users' likelihood of acting in a violent manner. Second, violent crimes may be committed by users to finance expensive drug habits. Third, crime may be related to the distribution and sale of drugs, particularly with respect to distributors' need to protect market share. Previous analyses of the US crack cocaine epidemic suggested that all three of these factors may have been involved in the crack cocaine epidemic, and in the escalation of violent crime in the US [4-7]. Suggestive evidence has been collected that the increase in crime may have been related to the relative youth of drug market participants [5]; the profitability of crack cocaine distribution and hence disputes over market share and distribution points [6]; and some contribution from the pharmacological effects of sustained crack cocaine use among low level user-dealers [7]. The relatively easy availability of firearms at that time has been argued to be a large contributor to the increases in homicide observed in the US during the period [5].

In contrast to the US, Australia has had relatively little notable cocaine use among problematic drug users [12-14]. In the later part of the 20^th ^century harms related to cocaine use have historically been low across the country [15,16]. This may be related to the high cost and relatively low availability of the drug in street based drug markets [12,16]; low rates of cocaine injecting or crack smoking among regular IDU [12,16]; and the purported concentration of use (because of its high costs) among smaller, advantaged social groups or commercial sex workers (who may have greater disposable income) [17-19].

In New South Wales (NSW) in the late 1990s, heroin was the drug most frequently reported by regular IDU as their drug of injection and choice. In early 2001, there were reports of a dramatic decline in the availability of heroin in Sydney, NSW [20,21]. This was confirmed by the 2001 Illicit Drug Use Reporting System (IDRS), Australia's strategic early warning system. The IDRS observed an overall reduction in the availability and street level purity of heroin, and an increase in heroin price for all major heroin markets that began in early 2001 and was sustained for much of that year [22,23].

Following this reduction in heroin supply, regular IDU reported less frequent heroin use, and more frequent cocaine use [24,25]. The availability of cocaine powder had also increased [24], although there was no evidence of the emergence of crack cocaine. An examination of changes in drug distribution in NSW suggested that those involved in street level and mid level heroin distribution began distributing cocaine when heroin became less available [26].

This sudden reduction in heroin use and increase in powder cocaine use provided a unique opportunity to conduct a natural experiment into the relationship between *powder *cocaine use and violent crime. We examined if changes in the nature or extent of violent crime in NSW following evidence of increased availability and use of powder cocaine were similar to those observed in New York when crack cocaine availability increased. Specifically, this paper aimed to do the following:

1. Examine changes in cocaine use in NSW from 2001 (see [24,26,27]);

2. Examine potential changes in rates of violent crime at this time;

3. Examine the extent to which these changes in cocaine use and crime were related.

## Methods

### Data used in the study

#### Semi-structured interviews with heroin users

Heroin users were recruited via advertisements placed in opioid pharmacotherapy clinics. They had to have (a) recent experience of the drug market and (b) to have commenced pharmacotherapy either between August and December 2000 (pre-shortage) or between February and April 2001 (during shortage). Fifty three users were interviewed in total, approximately half entering treatment in each time period. Users were surveyed on a range of issues including their involvement in and experience of drug markets prior to and during the heroin shortage [28].

#### Semi-structured interviews with key informants (KI)

Selection of key informants was based on one or more of the following:

• the extent of their contact with the illicit drug market;

• their level of knowledge of the illicit drug market and illicit drug users;

• the focus of their position (e.g. direct/indirect, operational/policy); and

• the length of time the key informant had held the position, particularly their ability to comment on changes over time, pre to post heroin shortage.

##### Law enforcement

The NSW Police Service comprises three levels of command: State, Region and Local Area Command (LAC). Key informants were selected across all three levels and across the four LAC responsible for policing the three Sydney open air drug markets, two region commands in which these LAC were located and a range of squads within the State Command (including squads focused on organised crime groups and drug crime).

A total of 22 law enforcement key informants were interviewed for this study, 20 of whom were sworn officers. Seven were state level personnel, 2 regional personnel and 13 LAC personnel. Seven interviewees held the position of Commander of their squad, 7 were managers of their unit, and 8 held general duty or operational positions (the latter includes 2 civilians – an analyst and a pharmacist).

##### Health

A total of 49 health KI were recruited for this study, including 5 from NSW State organisations. The remainder were recruited in the Sydney drug markets of Kings Cross (n = 16), Cabramatta (n = 14) and Redfern (n = 15). The roles of these KI were as follows: drug health (n = 25), community health (n = 3), community welfare (n = 5), emergency health (n = 1), indigenous health (n = 2), mental health (n = 2), prenatal health (n = 4), primary health care (n = 2) and youth services (n = 5).

#### NSW police incident data

NSW Police record all police activity in a centralised database known as the Computerised Operational Policing System (COPS). This information can be analysed at the level of 'event' or 'incident'. An *event *is a record created in COPS whenever police attend a criminal or non-criminal activity. An event includes the *incidents *that comprise it (what happened, where, who was involved) and the actions taken by police in response to the event. This information is not reliant on a charge having been laid (but offender details on gender and age may not be provided if the offender is not arrested). Information from this 'real time' dataset is downloaded at regular intervals for analysis by the NSW Bureau of Crime Statistics and Research (BOCSAR). The following incident types were used in the current study: cocaine possession/use, robbery with a firearm, robbery with a weapon (not a firearm), robbery without a weapon, homicide, assault, and weapons offences.

##### Time series analysis

**Figure 4:**
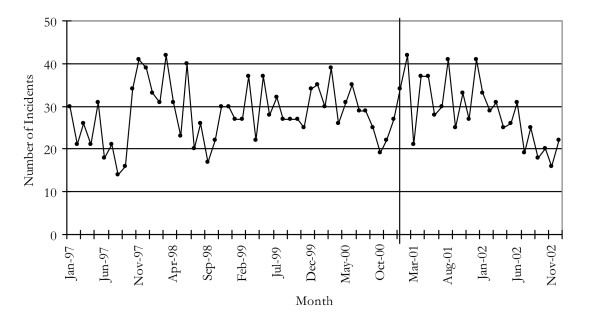
Incidents of homicide in NSW, 1997–2002

The indicator data series were analysed using an ARIMA model time series. The heroin shortage was represented in these models in the following three ways as: 1) a permanent effect (step); 2) a brief effect (pulse); or 3) a change in slope. Analyses dated the onset of the heroin shortage from January 2001, in accordance with the findings of other research on the course of the event [23]. Intervention models were fitted using SAS v 8.2. Intervention ARIMA models can require estimation of many parameters, and some of the data series lacked clearly definable responses at the point of the heroin shortage (e.g. Figure 4). In order to avoid large probability of type I error, analysis of data series which showed no evidence of a response to the heroin shortage on visual inspection were analysed by examination of crosscorrelation functions only. If the crosscorrelation functions for these series showed no clear evidence of an effect due to the shortage no further modelling was conducted on these series and the conclusion of no noticeable effect due to the heroin shortage was drawn.

## Results

### Trends in cocaine use

**Figure 1:**
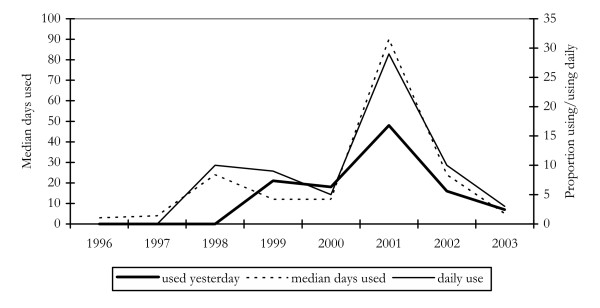
Proportion of IDU reporting cocaine use in the past six months, daily use, and use on the day preceding interview, 1996–2003

Clear increases were observed in the use of cocaine among regular injecting drug users in 2001 (Figure 1). This was true whether IDU were asked about their use of cocaine in the previous day, the number of days used in the past 6 months, or whether it was the last drug they had injected. This increase did not persist, however, with the proportion reporting cocaine use decreasing in 2002 and further in 2003 (Figure 1).

**Figure 2:**
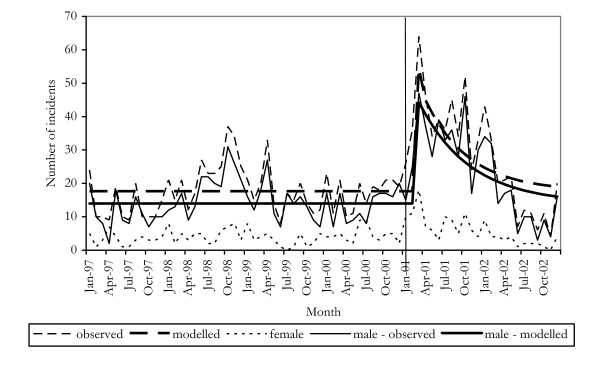
Incidents of cocaine possession/use in NSW, 1997–2002

Figure 2 shows the number of incidents recorded for cocaine possession/use in NSW. This peaked at 64 in March 2001 and remained high throughout the year but declined in 2002. The modelled series (Figure 2) showed that while police incidents for cocaine possession or use were at a steady level prior to the reduction in heroin supply, they increased significantly over the six months following the reduced heroin supply before returning to the levels seen prior to the heroin shortage. The maximum increase of 207% occurred 2 months after the shortage began (March 2001).

### Trends in robbery offences

**Figure 3:**
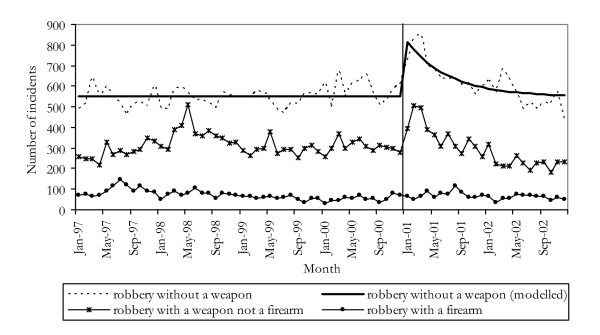
Incidents of robbery offences in NSW 1997–2002

Figure 3 shows the number of robbery offences in NSW for the period January 1997 to December 2002. The onset of the reduction in heroin supply was associated with a 33% (p < 0.0001) increase in the incidence of *robbery without a weapon*. The trend seen in incidents of *robbery with a weapon other than a firearm *followed a similar pattern. In contrast, there was no apparent effect of the heroin shortage upon the series *robbery with a firearm*.

Results of the time series analysis were consistent with the qualitative information collected in KI interviews. An increase in the incidence of robberies was the single most commonly reported change in criminal activity. KI consistently attributed it to a combination of the behavioural effects of cocaine and the need to increase criminal activity to fund the higher cost of using cocaine.

So, more likely to commit crime or for violence to be included in the crime and that seems to be likely given that people would be more desperate and presumably also, you know, if they are using cocaine in associated with their crimes, more reckless and more aggressive, abusive, volatile. (Health/Welfare KI)

KI reports described thefts 'gone wrong' in which excessive force and crude weapons were opportunistically used. Some drug users also reported attempting ill-planned armed robberies and being caught in the act by police

KIs reported that the type of crime engaged in by individual users during the heroin shortage was "out of character", and that users were less careful in the commission of crime. Overall, the tone of the offences changed: KIs and users in all markets reported that drug-related crime became more desperate, violent and impulsive. KIs reported that users stepped up their involvement in crime, moving from non-violent acquisitive crime (i.e. theft) to violent acquisitive crime (i.e. robbery).

The behavioural effects of cocaine meant that the execution of a theft often became more violent than intended or than was typical for that offender.

"The whole nature of the offences changed. There was no change in so far as people were still doing property offences, stealing – it's all the same. But people weren't getting enough money so they'd turn to violent offences. But not just that, because of the amount of cocaine they were using, it was just making them angry." (law enforcement KI)

### Trends in homicide and assault

**Figure 5:**
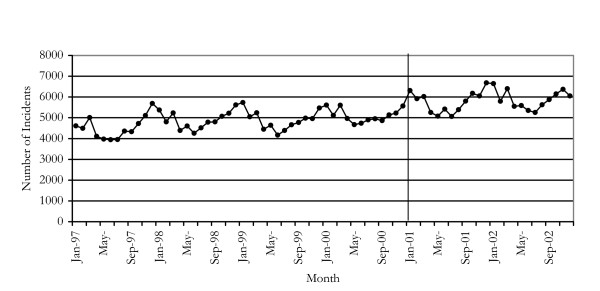
Incidents of assault in NSW, 1997–2002

Figures 4 and 5 show the incidence of homicide and assault offences between 1997 and 2002. There did not appear to be a change in either time series around the time when cocaine use increased. Apart from the general increase in violence commonly reported by KIs, there were no reports of any changes in the incidence of homicides and assaults.

### Trends in weapons offences

**Figure 6:**
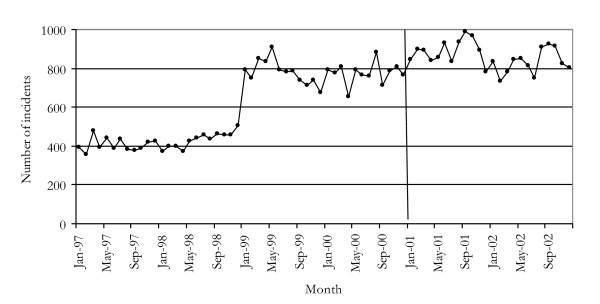
Incidents of weapons offences, NSW 1997–2002

Figure 6 shows the number of weapons offences in NSW. This offence category includes charges relating to the illegal possession, sale and discharge of firearms and offences relating to explosive/dangerous articles or threats. There was no change in the incidence of weapons offences at the time of the shortage (Jan–Apr 2001), either at a state or local level. It should be noted that the sharp increase in the series at the beginning of 1999 reflects a change in the legislation that gave NSW Police the power to conduct knife searches.

KIs typically mentioned the increased use of weapons in acquisitive crimes. Some thought that organised crime groups were involved in the distribution of firearms as part of their criminal repertoire, but these activities were not linked to either the reduction in heroin supply or the increase in cocaine availability and use.

## Discussion

This study has found a clear increase in the rate of violent crime concurrent with an increase in cocaine use. As has been shown elsewhere, there were marked increases in the use of cocaine among regular injecting drug users in major drug markets in Sydney [24,27]. There was also evidence from NSW Police records that this increase was observed at a State level for cocaine possession/use offences [26]. These findings were supported by data on the number of calls of concern to NSW telephone help lines about cocaine [27], and increased reports of cocaine as the last drug injected by NSP attendees [27],. The consistency of these changes suggests that there was a definite shift in drug use patterns in the IDU community from heroin to cocaine injecting.

These increases in cocaine use were accompanied by increased rates of violent crime. Consistent with the model proposed by Goldstein [11], interviews with KI of all types suggested that increases in violent acquisitive crime was related to *both *the psychopharmacological effects of heavy cocaine use, and also to the increased financial costs of users' drug use. KI reports were also obtained of violent crime occurring among those involved in cocaine distribution, but these could not be evaluated using police data.

Comparable research in other Australian States revealed little, if any, change in cocaine use among similar populations of IDU [29,30]. Furthermore, there was no significant increase in violent crime in these States. The absence of any increase provides further support for the argument that the increase in cocaine use among this disadvantaged group in NSW was causally related to the change in violent crime in that State. An increase in the rate of non-violent acquisitive crime in NSW [26] provided further evidence to support the notion that part of the increase in violent acquisitive crimes may have been related to the increased costs of drug users' habits following increases in the price of heroin, the previously dominant drug and the relatively higher cost of the cocaine that some switched to using after the onset of the heroin shortage.

Perhaps the most interesting difference between the experience in Sydney and New York with increasing cocaine use was the *lack *of any increase in the number of gun related incidents in Sydney, compared to a dramatic increase in such incidents that occurred in New York. There was an increase in the number of incidents of robberies involving weapons, but these did not involve guns. In New York, by contrast, many crimes involved guns and the homicide rate involving firearms increased markedly.

In New York and Sydney, organised crime groups have access to, and are involved in, the sale of illicit firearms. However, firearms have only limited availability for personal use in Australia [31], whereas they were relatively easily available in the United States at the height of the crack cocaine epidemic [5]. It seems possible that this availability leads to two sources of firearms in the US: licit sources, and illicit sources, whereby illicit sources may comprise the diversion of legally registered firearms as well as the large scale distribution of illegal firearms. In Australia, however, there are limited legal sources of handguns [31]. This suggests that maintaining such stringent controls upon firearms may have assisted in maintaining a low rate of firearm offences [32], *even in the face of increased cocaine use among criminally involved IDU that increased the risk of violent incidents*.

It is necessary to consider the potential influence of the forms of cocaine used in Australia, compared to the US and UK. In Australia, there is little or no use of "crack" cocaine in the country [12,33]; in contrast, the concerns related to cocaine use in the UK and US have largely centred on harms related to apparent epidemics of "crack" cocaine use. However, the current study has found an association between the increased use and availability of cocaine *powder *and violence, suggesting that the increased availability and use of cocaine powder may have a similar impact on violent crime as crack cocaine.

## Limitations

This paper is subject to the flaws that beset all natural experiments, in that it is not possible to guarantee that the intervention being studied was the only event that affected cocaine use and/or violent crime in the time period. However, similar research on rates of crime conducted in the same time period in both Victoria and South Australia provided a control series. These two states were geographically isolated from NSW and both experienced a heroin shortage but neither experienced any increase in cocaine use among IDU.

Although it might be possible that some other event interfered in NSW drug markets at the same time as the heroin shortage, such possibilities were examined in a process of extensive crosschecking through KIs, consultation with stakeholders and analysis of other data sources in the wider project from which this study is drawn. No plausible alternative explanations remained [34,35].

Another limitation of the analysis concerns the relative simplicity of the analyses we have conducted. Ideally, it would be of interest to model trends over time in violent acquisitive crime that include not only cocaine use, but also other factors such as risk of arrest for robbery, the proportion of those committing robbery offences who were imprisoned, and the rate of unemployment in the community. As a result of reduced heroin availability, police reported having greater resources (in terms of available personnel) to target drug dealers involved in distributing drugs other than heroin, but it was consistently reported by police to be the case that those involved in low-level heroin distribution switched to cocaine distribution [26].

The increase in availability and use of cocaine was relatively short-lived, which was driven largely by a lack of cocaine available to sustain the 2001 levels [16]. Likewise, the increase in violent crime was also short-lived, thereby adding to the case for it being a causal factor in the increased rate of violent offences. It is unknown what the consequences would have been if the increased cocaine supply and use had persisted for a much longer period of time.

This natural experiment provided a unique opportunity to identify the effects of a sudden increase in cocaine use in a major Australian drug market, and to investigate previous findings regarding the role of cocaine use in violent criminal activity. Given the extensive attempts to eliminate other causes of the increase in violent crime and the existence of a partial control group, it seems reasonable to conclude that a transient increase in cocaine use among IDU in New South Wales produced a transient increase in violent crime.

## Conclusion

Increases in cocaine use in NSW were accompanied by increases in violent crime as were observed in New York in the 1980s. However, these violent crimes did *not *involve the use of firearms, providing some supporting for the value of stringent gun control laws in reducing access to guns by criminals [32].

## Competing interests

The author(s) declare that they have no competing interests.

## Authors' contributions

LD & CD conceived of the study, and participated in its design and coordination and helped to draft the manuscript. WH drafted the manuscript. EC carried out the data collection and drafted the manuscript. SG performed the statistical analysis. All authors read and approved the final manuscript.

## Pre-publication history

The pre-publication history for this paper can be accessed here:


